# Use of Spinal Anesthesia in Pediatric Laparoscopic Appendectomies: Case Series

**DOI:** 10.2196/25204

**Published:** 2021-04-28

**Authors:** Md Jafrul Hannan, Mosammat Kohinnor Parveen, Alak Nandy, Md Samiul Hasan

**Affiliations:** 1 Department of Pediatric Surgery South Point Hospital Chittagong Bangladesh; 2 Department of Pharmacology & Therapeutics Rangamati Medical College Rangamati Bangladesh; 3 Department of Anesthesiology Chattagram Maa-O-Shishu Hospital Medical College Chittagong Bangladesh; 4 Department of Pediatric Surgery Dhaka Shishu Hospital Dhaka Bangladesh

**Keywords:** pediatrics, appendectomy, spinal anesthesia, general anesthesia, laparoscopy, vomiting, keyhole, surgery, anesthesia, appendix

## Abstract

**Background:**

Owing to the widespread use of general anesthesia, administration of spinal anesthesia in pediatric patients is not widely practiced. Yet there is ample positive evidence demonstrating its safety, effectiveness, and success.

**Objective:**

The objective of this study is to compare postoperative patient comfort, length of hospital stay, and cost-effectiveness of pediatric laparoscopic appendectomies performed under spinal and general anesthesia with the usual standard-of-care procedures employed in the hospital.

**Methods:**

This is a case series of 77 consecutive pediatric laparoscopic appendectomies (involving 5-8–year-old children) that took place in a hospital in Chittagong, Bangladesh, in 2019. A total of 40 patients underwent spinal anesthesia and 37 patients underwent general anesthesia. Variables such as surgery and operation theater times, pain score, incidence of postsurgery vomiting, analgesic usage, discharge times, and hospital costs were recorded. Statistical analysis was used to analyze the data as a function of anesthesia type.

**Results:**

The probability of vomiting when using spinal compared to general anesthesia was lower within the first 5 hours (*P*<.001) and 6 hours (*P*=.008) postoperation. A significant difference (*P*<.001) was observed between the total costs of the two procedures, with spinal anesthesia being less expensive. Patients were more likely to be discharged the same day of the procedure when spinal anesthesia was used (*P*=.008).

**Conclusions:**

Spinal anesthesia has many advantages compared to general anesthesia for pediatric laparoscopic appendectomies. Patient comfort is improved due to a significant decrease in vomiting. This allows for more rapid hospital discharges and substantial cost savings, without compromising the outcome of the procedure.

## Introduction

The history and success of pediatric spinal anesthesia procedures, beginning with the 1898 report by Bier and several studies by Gray and Cantab a few years later, has recently been documented [1]. Due to improvements in general anesthesia, there was little interest in pediatric spinal anesthesia until the 1950s, when more studies advocated for its use in children [2]. Since then, the spinal anesthetic approach has increased dramatically in children, and the potential problems and risks of general anesthesia in pediatrics have been documented [3]. However, even by 1984, Abajian et al [4] noted that despite reports of spinal anesthesia use in children and confirmation that it is a safe alternative to general anesthesia even for patients under 1 year of age, it remained underutilized. In 2006, Williams et al [5] found complication rates to be very low among 1554 procedures and recommended spinal anesthesia for lower abdominal or extremity surgery in infants. An Italian and Finnish collaborative published a study of 1132 children, aged 6 months to 14 years, with similar conclusions (specifically with hyperbaric bupivacaine) [6]. Imbelloni et al [7] reported an excellent rate of success in 307 consecutive cases of patients under the age of 13 years in a Brazilian setting, although they cautioned that spinal anesthesia in children should be administered only by anesthesiologists already trained in spinal anesthesia in adults. They further noted that the cost to the facility was 54% less than the cost of general anesthesia, which is an important consideration in countries with limited financial resources. In Nigeria, even as recently as 2010, only general anesthesia was used. The first study in Nigeria indicated that spinal anesthesia in children caused minimal hemodynamic disruption and was classified as a safe technique for lower-extremity surgeries [8]. In 2010, Polaner and Drescher [9], and a year later Ecoffey [10], reviewed the safety record and concluded that although usage of regional anesthesia, whether as adjuncts, primary anesthesia, or postoperative analgesia, was becoming increasingly common in pediatric practice, data on their safety remained limited because of the scarcity of large-scale prospective studies required to detect low-incidence events. Despite this, their study concluded that regional blockades in infants and children appeared to have a very high degree of safety. They noted the importance of attention to technique, detail, and prudent patient selection to avoid possible complications.

Despite these positive outcomes, even as recently as 2018, there have been some debate regarding pediatric spinal anesthesia. The European Society of Regional Anesthesia and Pain Therapy and the American Society of Regional Anesthesia and Pain Medicine published their recommendations on local anesthesia and adjuvant dosage in pediatric regional anesthesia, conspicuously noting that up to that point there was a large variability of dosages used in clinical practices. Their recommendations were intended to curb that variability [11]. The technique is still gaining traction, and even as recently as 2019, its benefits have been again summarized [12]. A recent report out of Pakistan [13] noted the successful use of spinal anesthesia in surgeries for the past 20 years, with the only real danger being when it was applied by poorly or untrained personnel.

Another recent area of debate is the applicability of spinal anesthesia to laparoscopic approaches to surgery. One of the first reports of laparoscopy under spinal anesthesia was reported by Islam et al [14] in 2014, where laparoscopic pyloromyotomy procedures in infants were investigated. Of the 12 cases studied, 9 were successful, while the other 3 cases required conversion to general anesthesia. The 3 failures were related to the inability to access the intrathecal space and an inadequate block level so that the infant did not tolerate insufflations of the abdomen. More recently, Chiao and Boretsky [15] presented 3 case reports employing laparoscopic surgery for inguinal hernia repair. All procedures were successful, with 1 patient experiencing hypertension and tachycardia during insufflations with brief supplemental use of sevoflurane. The authors concluded that the use of spinal anesthesia for laparoscopic surgery was successful, with the advantage of decreased exposure to opioids and general anesthesia agents, some of which are potential neurotoxins that may negatively affect brain development. This can provide an additional anesthesia option for providers and families. The authors claimed that laparoscopy could, perhaps, no longer be viewed as being incompatible with the use of spinal anesthesia in infants.

Despite the increased prevalence and positive outlooks of spinal anesthesia in children, it is still not practiced everywhere owing to the widespread use of conventional general anesthesia. In this paper, we present a case series of 77 consecutive pediatric laparoscopic appendectomy patients, comparing their postoperative comfort (measured by the incidence of vomiting in the postoperative period), length of hospital stay, and cost-effectiveness of the procedure performed under spinal and general anesthesia.

## Methods

### Overview

This case series of 77 consecutive pediatric (5-8–year-old children) laparoscopic appendectomies took place at South Point Hospital, Chittagong, Bangladesh, between January 1 and December 31, 2019. Anesthesia choices were not predetermined but decided during the operation. Those receiving spinal anesthesia (n=40) also received sedation with diazepam or ketamine hydrochloride injection as an adjunct to alleviate their anxiety and help them remain calm. Patients who received general anesthesia (n=37) also received nitrous oxide gas throughout the intraoperative period as analgesics and were kept relaxed by rocuronium. These represent the current standard of care for these procedures at the hospital.

Spinal anesthesia consisted of 0.5% bupivacaine in 8.5% dextrose at a dose of 0.4 mg/kg of body weight. CO_2_ insufflations pressures were kept under 8 mmHg, and the flow was maintained between 2.0-2.5 L/min. For all procedures, irrespective of the type of anesthesia, antiemetics were administered at the start of the procedure, while dosages of NSAIDs (nonsteroidal anti-inflammatory drugs) were administered toward the end of the operation, per the usual practice in hospitals. Feeding was recommenced 4-5 hours postoperation for the general anesthesia group and 2-3 hours postoperation in the spinal anesthesia group.

The ethical clearance for this study was provided by South Point Hospital (Admn/SPH/191/2020).

### Hypotheses

We hypothesized that spinal anesthesia is better than general anesthesia for pediatric patients in terms of postoperative comfort and cost-effectiveness. Our null hypotheses were as follows: probability of vomiting <5 hrs postoperation is greater for spinal than general anesthesia; probability of vomiting >6 hrs postoperation is greater for spinal than general anesthesia; and probability of same-day discharge is greater for general than spinal anesthesia.

### Statistical Analyses

Statistical analysis of the data was performed using JMP statistical software (SAS Institute). Significance was held at the 95% level unless otherwise noted (minimum 90% level). Chi-square and Fisher exact tests were used for contingency analysis of categorical data. Parametric (Student *t* tests) or nonparametric tests (Wilcoxon) were used for comparison of continuous numerical data depending on the normality of the data, determined using the Shapiro-Wilk test. The effects of anesthesia on vomiting during the first 5 hours postoperation and after 6 hours postoperation, time until patient discharge, and cost of the procedure were examined.

Finally, all factors were combined in a multiple correspondence analysis. Multiple correspondence analyses are the categorical equivalent of principal component analysis in multivariate statistics. It produces a plot, which is a 2D representation of “n-space,” where *n* is the number of variables. The 2 dimensions chosen are those that explain the most variance in the data. The closer the points are to this plot, the more highly they are associated with one another on a relative basis, while the further away from the origin the points are located, the more they are discriminating themselves from the rest of the data.

### Data Availability Statement

The data sets generated during and/or analyzed during the study are available on Figshare [16].

## Results

The descriptive statistics for the cohort of 77 patients in the series are presented in Table 1. The data indicate an approximate even distribution of patients across gender, age, and anesthesia method used for the procedure.

Results pertaining to incidence of vomiting up to 5 hours and after 6 hours postoperation are provided in Table 2. The odds ratios (ORs) for the incidence of vomiting based on administration of general anesthesia are also provided with 95% confidence limits.

For the case of <5 hours postoperation, the *P* values determined by the Fisher exact test were all less than .05 for the entire cohort as well as when stratified by gender and age. The null hypothesis was therefore rejected, and the probability of vomiting was determined to be greater when general anesthesia was used. The odds for vomiting within the first 5 hours postoperation when general anesthesia was used for the overall cohort was 8.1, with males exhibiting a maximum OR of 15.6 and females exhibiting a minimum OR of 4.4.

After 6 hours postoperation, the same null hypothesis was only rejected for the entire cohort, females, and the younger age bracket of 5-6–year-old patients. The OR spread for these 3 cohorts is less compared to the first 5 hours postoperation (OR 3.5, 5.7, and 5.0, respectively).

**Table 1:** Descriptive statistics of the patient cohort by gender, age, and anesthesia type used for the procedure (N=77).

Characteristic		Count, n (%)
**Gender**		
	Female	38 (49.4)
	Male	39 (50.6)
**Age (years)**		
	5	17 (17.2)
	6	17 (17.2)
	7	24 (24.3)
	8	19 (19.3)
**Anesthesia type**		
	Spinal anesthesia	40 (52.0)
	General anesthesia	37 (48.0)

**Table 2:** Statistical analysis of the effect of anesthesia on incidence of vomiting up to 5 hours postoperation and after 6 hours postoperation. The odds of vomiting when a general anesthetic was used is given with the 95% upper and lower confidence limits.

Null hypothesis and cohort			*P* value^a^	Odds ratio (95% confidence limits)
**Probability of vomiting <5 hrs postoperation is greater for spinal than general anesthesia**				
	All		<.001	8.1 (2.9-22.4)
	**Gender**			
		Male	<.001	15.6 (3.2-77.2)
		Female	.04	4.4 (1.1-17.8)
	**Age (years)**			
		5-6	.02	6.7 (1.4-32.3)
		7-8	<.001	13.0 (2.9-58.9)
**Probability of vomiting >6 hrs postoperation is greater for spinal than general anesthesia**				
	All		.008	3.5 (1.4-9.3)
	**Gender**			
		Male	.17	2.4 (0.62-9.0)
		Female	.02	5.7 (1.4-23.5)
	**Age (years)**			
		5-6	.04	5.0 (1.1-23.2)
		7-8	.12	2.6 (0.73-9.0)

^a^Fisher exact test.

The effect of anesthesia type on hospital discharge is summarized in Table 3. The ORs for same-day discharge were calculated based on the administration of spinal anesthesia. The *P* values from the Fisher exact test rejected the null hypothesis for the entire cohort, as well as for the female group and the younger age bracket. Thus, the probability of same-day discharge was greater when spinal anesthesia was used. This mirrors the result for the probability of vomiting after 6 hours postoperation. The OR values indicate that the younger age brackets were particularly more likely to be discharged on the same day when spinal anesthesia was used compared to the overall cohort (OR 6.8 vs OR 3.5).

**Figure 1:**
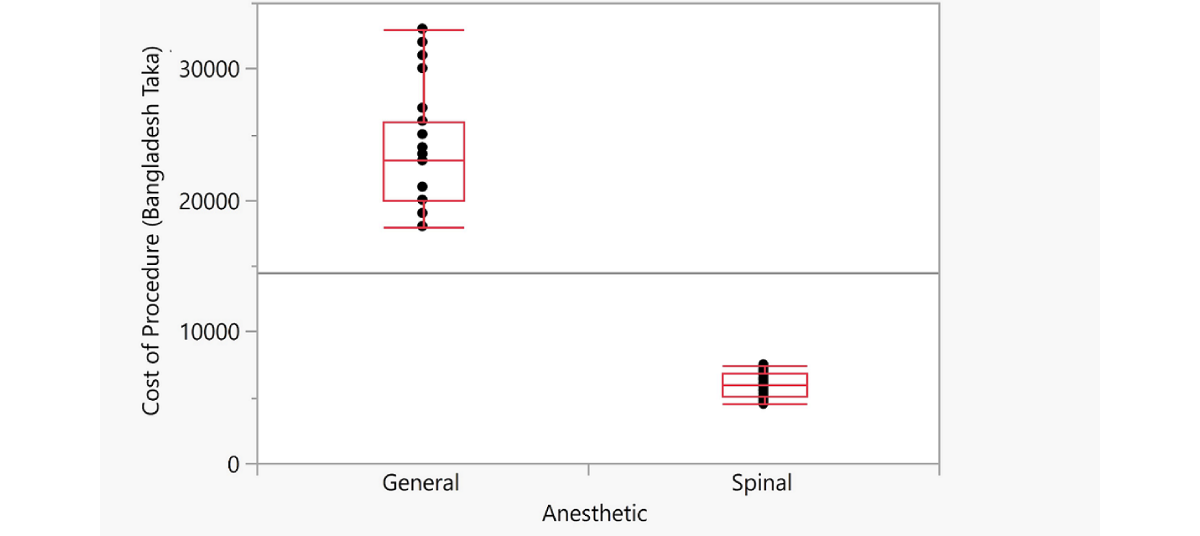
Comparison of the cost of laparoscopic appendectomies between procedures with general and spinal anesthesia (in Bangladesh taka; 1 USD=84.75 BDT).

A comparison of the cost of the procedure (in Bangladesh taka; 1 USD=84.75 BDT) when the different types of anesthesia were used is shown by the box plots in Figure 1. Results from the Shapiro-Wilk tests indicated that the data did not follow a normal distribution and thus a Wilcoxon test was used to test for a significant difference. The *P* value calculated was <.001, indicating that the costs encountered when using spinal and general anesthesia were significantly different. Use of spinal anesthesia was less expensive.

The effects of the adjuncts diazepam and ketamine hydrochloride on the spinal anesthesia group were also examined in terms of incidence of vomiting, but no significant differences were found up to 5 hours postoperation (Fisher exact test, two-tailed; *P*=.26) or after 6 hours postoperation (*P*=.48). These adjuncts also did not affect the cost of the procedure (Student *t* test; *P*=.26) nor the speed of discharge (Fisher exact test, two-tailed; *P*=.48).

**Figure 2:**
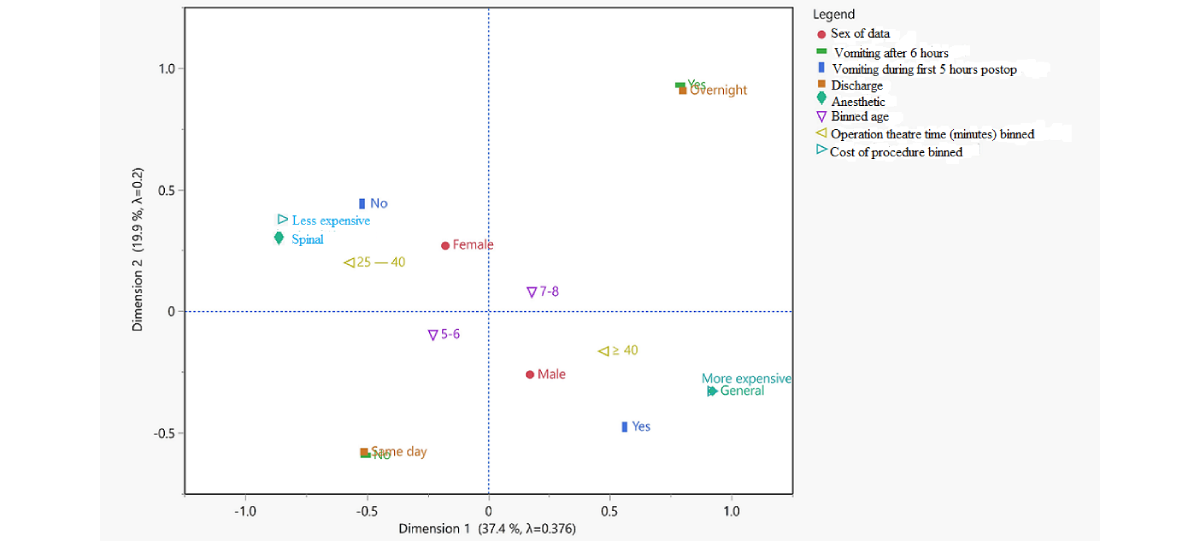
Multiple correspondence analysis plot from variables listed.

For the multiple correspondence analyses, the operation time and the theater time were binned into two categories: above and below the median value. The cost of the procedure was binned into “less expensive” (less than 15,000 Bangladesh taka) and “more expensive” (greater than 15,000 Bangladesh taka) categories. The resultant plot is shown in Figure 2.

The plot of these 2 dimensions explains 57% of the variance in the data and shows astonishingly well how “less expensive” and spinal anesthesia are associated (they lie practically on top of each other). Other factors found to be associated with the “less expensive” category included an operation theater time between 25-40 minutes (the shortest time bin), no vomiting during the first 5 hours, and female patients.

**Table 3:** Statistical analysis of the effect of anesthesia type on hospital discharge. The odds ratio of same-day hospital discharge when spinal anesthesia was used is provided with 95% upper and lower confidence limits.

Null hypothesis and cohort			*P* value^a^	Odds ratio (95% confidence limits)
**Probability of same-day discharge is greater for general than spinal anesthesia**				
	All		.008	3.5 (1.4-9.3)
	**Gender**			
		Male	.11	2.9 (0.75-10.9)
		Female	.04	4.4 (1.1-17.8)
	**Age (years)**			
		Age 5-6	.02	6.8 (1.4-32.4)
		Age 7-8	.18	2.2 (0.62-7.6)

^a^Fisher exact test.

## Discussion

Laparoscopic surgery is now the method of choice for lower abdominal procedures. Childers et al [17] reported that of the 9507 appendectomies conducted in children under the age of 18 years in the United States, 94.6% used laparoscopy. In 4 central European institutions, of the 519 pediatric appendectomies performed, 79.6% were conducted via laparoscopy [18]. In Germany, Gosemann et al [19] found that of 8110 pediatric appendectomies, 75% were performed using laparoscopy. In 2018, in a wide-ranging study, Tom et al [20] found that of the 58,511 appendectomies conducted in children’s hospitals in the United States between 2003 and 2012, 70% were done using laparoscopy, compared to 53% of the ~1.2 million conducted at nonchildren’s hospitals. Zani et al [21] summarized the results of the European Pediatric Surgeons’ Association survey on the management of pediatric appendicitis, compiled from 169 respondents from 42 countries (24 European countries). For simple appendicitis, laparoscopy was the preferred method for 89%, while for perforated appendicitis, it remained the method of choice for 81%. In Japan, Fujishiro et al [22] found that of the 4489 pediatric appendectomies performed, 70.5% were performed laparoscopically. It is clear from these studies that for pediatric appendicitis, laparoscopy is the method of choice, which was also the conclusion of a review of pediatric appendicitis by Rentea et al [23]. However, in all of these studies, an important fact is conspicuously absent. No mention of the type of anesthesia administered during the procedure is provided. An Egyptian study of 390 complicated pediatric appendicitis cases was published by Khirallah et al [24], comparing laparoscopic (200 cases) and open appendectomies. All procedures were conducted under general anesthesia, and the authors concluded that the laparoscopic technique should be pediatric surgeons’ first choice for appendectomy procedures. Thus, our study clearly addresses a paucity of data pertaining to the effect of the type of anesthesia on pediatric laparoscopic procedures in terms of postoperative patient vomiting, discharge time, and relative costs.

The results of the present study clearly showed that the use of spinal anesthesia reduced the likelihood of vomiting during both the first 5 hours and after 6 hours postoperation (Table 1). This mirrors the results of Verma et al [25] in their study of 102 pediatric patients aged 6 months to 14 years undergoing various surgeries, including herniotomy, appendectomy, genitourinary surgeries, and lower limb orthopedic surgeries, under spinal anesthesia. In this cohort, no incidence of vomiting was noted. Similarly, Ahmed et al [26] in their study of 78 children with a similar range of procedures reported 6 cases of nausea and 1 case of vomiting. Kokki and Hendolin [27] reported 10 patients experiencing nausea but no vomiting in a cohort of 52 patients between the ages of 7 and 18 years undergoing lower umbilical procedures with spinal anesthesia (bupivacaine in 8% dextrose). None of these studies stratified the incidence of vomiting by gender, so in that respect, the results of our study are, to the best of our knowledge, novel. However, the studies by Verma et al [25] and Ahmed et al [26] were largely male dominated (>80%); therefore, our observation that males are especially less likely to experience vomiting in the first 5 hours postoperation is not unexpected. Nonetheless, the entire subject of postoperative nausea and vomiting can be quite complex [28].

There is ample evidence for shorter hospital stays with a laparoscopic procedure [19,29-31] although the study by Fujishiro et al [22] contradicted this observation. They found no significant difference between laparoscopic and open appendectomies in terms of length of stay. The present results showed a definite trend for overnight stays when general anesthesia was used, whereas same-day discharges were highly associated with spinal anesthesia (Table 3).

Teja et al [32] have championed the need for more cost-effectiveness research in anesthesiology. They noted a paucity of cost-effectiveness data, particularly from a pediatric perspective. Although the research to this end is relatively simplistic and relates only to the cost of the procedures, a significant reduction in cost (by nearly a factor of 5; Figure 1) was found in this study when spinal anesthesia was used. Imbelloni et al [7] reported a savings in anesthesia cost of 54% when the spinal method was used compared to historical data. This was pooled using a variety of pediatric procedures.

In summary, the results of this case series provide a clear indication that spinal anesthesia has advantages to general anesthesia in laparoscopic appendectomy procedures. The data provided strong evidence for more rapid hospital discharges and substantial cost savings, without compromising the outcome of the procedure and postoperative comfort of the patient.

## Abbreviations

NSAID: nonsteroidal anti-inflammatory drug
OR: odds ratio
